# Usefulness of time-resolved MR angiography in spinal dural arteriovenous fistula (SDAVF)—a systematic review and meta-analysis

**DOI:** 10.1007/s10143-023-02242-7

**Published:** 2023-12-11

**Authors:** Katarzyna Wójtowicz, Lukasz Przepiorka, Edyta Maj, Sławomir Kujawski, Andrzej Marchel, Przemysław Kunert

**Affiliations:** 1 Department of Neurosurgery, Medical University of Warsaw, Warsaw, Poland; 2 Second Department of Clinical Radiology, Medical University of Warsaw, Warsaw, Poland; 3 Department of Exercise Physiology and Functional Anatomy, Ludwik Rydygier Collegium Medicum in Bydgoszcz Nicolaus Copernicus University in Toruń, Bydgoszcz, Poland

**Keywords:** Magnetic resonance angiography, Spinal dural arteriovenous fistula, Digital subtraction angiography, Diagnosis

## Abstract

Spinal dural arteriovenous fistulas (SDAVFs) constitute the most common type of spinal vascular malformations. Their diagnosis requires spinal digital subtraction angiography (DSA), which is time-consuming, requires catheterizing many vessels, and exposes patient to a high radiation and contrast doses. This study aims to evaluate the usefulness of time-resolved MR angiography (TR–MRA) in SDAVF diagnosis. We performed a systematic review of the PubMed and EMBASE databases followed by a meta-analysis. TR–MRA was an index test, and spinal DSA was a reference. Of the initial 324 records, we included 4 studies describing 71 patients with SDAVFs. In 42 cases, TR–MRA was true positive, and in 21 cases, it was true negative. We found 7 false-positive cases and 1 false negative. TR–MRA allowed for shunt level identification in 39 cases. Of these, the predicted level was correct in 23 cases (59%), to within 1 level in 38 cases (97.4%) and to within 2 levels in 39 cases (100%). The diagnostic odds ratio was 72.73 (95% CI [10.30; 513.35]), *z* = 4.30, *p* value < 0.0001. The pooled sensitivity was 0.98 (95% CI [0.64; 1.00]), and the pooled specificity was 0.79 (95% CI [0.10; 0.99]). The AUC of the SROC curve was 0.9. TR–MRA may serve as a preliminary study to detect SDAVFs and localize the shunt level with sensitivity and specificity as high as 98% and 79%, respectively. Unless the TR–MRA result is unequivocal, it should be followed by a limited spinal DSA.

**Supplementary Information:**

The online version contains supplementary material available at 10.1007/s10143-023-02242-7.

## Introduction

Spinal dural arteriovenous fistulas (SDAVFs) constitute the most common type of spinal vascular malformations. They present with nonspecific symptoms and lead to paraplegia and sphincter dysfunction. Successful treatment may reverse such disastrous symptoms—hence, the need for prompt diagnosis [1, 2].

The diagnosis of SDAVF requires spinal digital subtraction angiography (DSA). Spinal DSA is a time-consuming study that requires catheterizing many vessels, exposes the patient to high radiation and contrast doses, but remains the gold standard. Recently, some researchers reported time-resolved magnetic resonance angiography (TR–MRA) as a useful tool in SDAVF diagnosis.

Time-resolved magnetic resonance angiography is a relatively new MR technique (introduced by Schoenberg et al. in 1999 [3]) which is able to obtain images of vessels in subsequent phases of enhancement after contrast agent administration and thus provide hemodynamic information by visualizing the passage of the contrast through arterial and venous vessels. Advanced MR systems enable the shortening of the acquisition time of each phase to as low as 1–2 s while increasing the temporal resolution. Thus, this enables the imaging of arterial inflow without overlapping veins, which is especially important in the case of such pathologies as SDAVF, in which the inflow into the venous vessels is rapid. However, TR–MRA’s sensitivity, specificity, and accuracy in locating shunt remain unexamined.

This study aims to evaluate the usefulness of TR–MRA in the diagnosis of SDAVF by determining its sensitivity, specificity, and accuracy in locating fistulas. Until now, no one has addressed this question in a systematic review. The significance of this research lies in the fact that such a noninvasive study could improve the diagnostic process of SDAVFs and provide an additional tool in cases of unexplained myelopathy. Furthermore, an initial TR–MRA could greatly facilitate a subsequent spinal DSA by reducing the number of catheterized vessels, as well as the radiation and contrast dose.

## Methods

### Search strategy and selection criteria

This review follows the PRISMA 2020 guidelines for systematic reviews [4]. We designed and conducted a comprehensive literature search of the PubMed and EMBASE databases. We looked for eligible studies from the inception of each database to October 2022 based on the following phrases: (spinal dural arteriovenous fistula) AND (“Magnetic Resonance Angiography”[Mesh]) for PubMed and analogical for EMBASE; we did not use additional filters.

Our diagnosis of interest was dorsal intradural spinal vascular malformation according to the Spetzler classification [5]. TR–MRA was an index test and spinal DSA was a reference. In selected cases, we considered unequivocal intraoperative findings (and not a spinal DSA) as conformation of SDAVF. When clinical outcomes were evident for the absence of a SDAVF, we characterized such cases as true negatives.

We included studies published in English that reported patients with a suspicion of SDAVF that was based on clinical and radiological evaluation. SDAVFs have characteristic appearance in standard contrast-enhanced spinal MRI: hyperintense signal of spinal cord in T2 sequence and intradural dilated and tortuous vessels [6, 7]. We excluded studies performed in populations with already confirmed SDAVFs (when SDAVF has been diagnosed and TR–MRA was used afterwards, e.g., for research purposes or to gain experience in TR–MRA). We also excluded studies that disregarded TR–MRA results in patients with initial suspicion of SDAVF that was subsequently excluded in DSA. In short, our inclusion and exclusion criteria were as follows:

- Papers that reported patients with a clinical or radiological suspicion of SDAVF, in which TR–MRA was performed first, and then spinal DSA (or surgery) served as confirmatory tests.
- Papers that failed to report the results of both studies (TR–MRA and spinal DSA) in each patient.

**Fig. 1:**
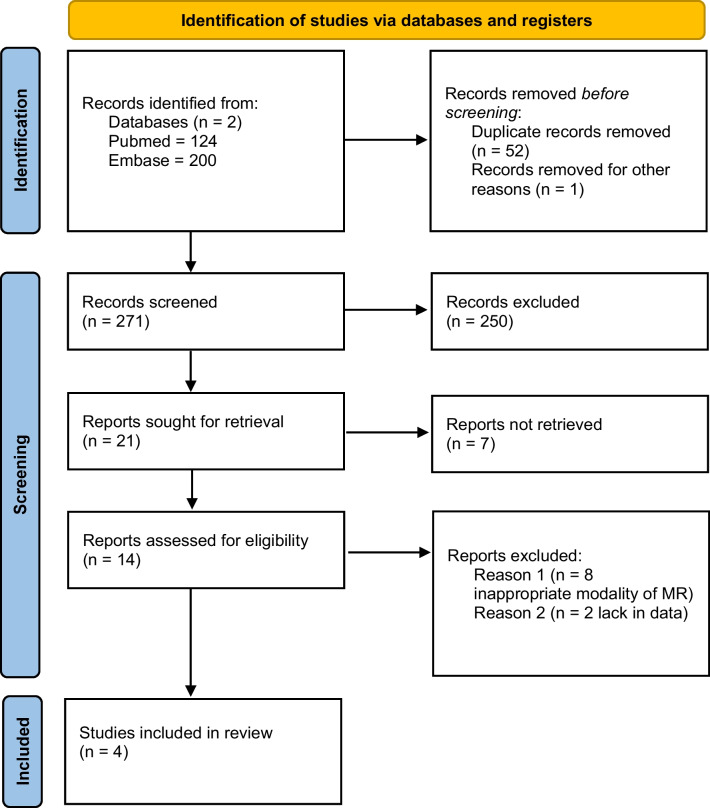
Literature review search strategy. MR, magnetic resonance

Two independent reviewers screened 272 titles and abstracts and then read the full text of the remaining 21 articles. Seven articles were excluded because the authors reported their experience in SDAVF diagnosis with noninvasive imaging techniques; those were not studies that evaluated population with SDAVF suspicion. We excluded 8 further studies because different imaging modalities were used. Two other studies initially had seemed to have met the inclusion criteria but were later excluded as described later in the text. Disagreements were resolved by seeking the opinion of the experienced neuroradiologist (Fig. 1).

### Data extraction

We designed a data extraction form that was filled by two reviewers for each accepted study, and it included population, study design (randomized or nonrandomized, prospective or retrospective), year of publication, number of patients, number of nondiagnostic TR–MRA exams, sensitivity, specificity, positive and negative predictive values, and accuracy in locating fistula level (Table 1). Table 2 presents selected TR–MRA parameters [magnetic field strength of MR scanner, repetition time (TR), echo time (TE), flip angle, FOV, type and dose of contrast, temporal update, slice thickness, and number of postcontrast phases]. The experienced neuroradiologist evaluated the TR–MRA protocols.

**Table 1:** Included studies

Author and year	SDAVF number (%) out of all patients	Sensitivity	Specificity	PPV	NPV	Precision in fistula level prediction
Ali et al., 2007 [14]	3 (27.3%)	100%	100%	100%	100%	100% within 1 level
Saindane et al., 2014 [15]	8 (44.4%)	88%	90%	88%	90%	85.7% within 1 level
Mathur et al., 2017 [16]	10 (66.7%)	100%	80%	91%	100%	100% within 1 level
Naamani et al., 2022 [17]	22 (81.5%)	100%	0%	71.4%	NA	50% in predicting the exact level of the SDAVF, 90% within 1 level

*PPV* positive predictive value, *NA* not applicable, *NPV* negative predictive value, *SDAVF* spinal dural arteriovenous fistula

**Table 2:** Summary of time-resolved magnetic resonance angiography (TR–MRA) techniques

Study	T	TR [ms]	TE [ms]	Flip angle	Contrast	Contrast dose	FOV [cm]	Post contrast phases	Temporal update [s]	Slice thickness [mm]
Ali et al., 2007 [14]	1.5	2.8	1.2	20	Gadopentetic acid	15 ml	No data	40–44	2.32–6.75 s	1.6–2.5 mm
Saindane et al., 2014 [15]	1.5	2.54	1.97	35	Gadobenic acid	0.1 mmol/kg	35	10	7 s	2 mm
Mathur et al., 2017 [16]	1.5	3.5–4	1–1.5	35	Gadobutrol	10 ml	30	20	2.2 s	3 mm
Naamani et al., 2022 [17]	1.5	2.40	No data	No data	Gadobutrol	20 ml	32	No data	2.4 s	3 mm

*FOV* field of view, *T* tesla, *TE* echo time, *TR* resonance time

The outcomes of this study were TR–MRA sensitivity, specificity, positive and negative predictive values, and accuracy in locating shun level in SDAVF diagnosis. We used the Quality Assessment of Diagnostic Accuracy Studies–2 (QUADAS–2) tool for assessing the quality of diagnostic accuracy [8].

### Statistical analysis

We estimated from each study means and 95% confidence intervals (CIs) for sensitivity and specificity and presented them with paired forest plots. The Wilson method was used to calculate CI.

The mean age of patients from two of the four analyzed studies was estimated based on median values using a method described by Hozo et al. [9]. We created summary receiver-operating characteristic (SROC) curves with false-positive rate on the *x*-axis and sensitivity on the *y*-axis. We used paired forest plots and SROC curves to provide a subjective assessment of the presence or absence of heterogeneity. The bivariate model estimated the summary parameters sensitivity and specificity of the included studies. Continuity correction of 0.5 was used, if applicable. Heterogeneity between studies was assessed using *I*^2^ (25–49% was considered to be low heterogeneity, 50–74% was moderate, and > 75% was high heterogeneity). However, as *I*^2^ can be biased in small meta-analyses, we have reported the results of both random and fixed models in all comparisons [10].

We did not perform the sensitivity analysis. To conduct meta-analysis, R environment with mada, meta, metafor, and metavix packages was used [11]. We did not use automation tools in our review. We did not need to gather additional information from study investigators with the exception to the studies by Amarouche et al. and Kannath et al. [12, 13]. In these cases, however, with no response, the studies were eventually excluded (as described in detail below).

## Results

### Literature search results

This systematic review included 4 studies, summarized in Table 1. Risk of bias was assessed with QUADAS–2 tool (On–line Table 1).

In this systematic review, we included 71 patients with distinctive clinical presentation or MR findings suggestive of SDAVFs. Study sizes ranged from 11 to 27 patients. Seventy-six percent (54/71) of patients were male; mean age of patients was 61 years old, range 24–91. We identified 43 cases of SDAVFs, which were diagnosed with spinal DSA (in 41 cases) or intraoperatively (in 2 cases)—when spinal DSA failed to present SDAVF. In the remaining 28 cases, SDAVFs were excluded: in 24 cases with spinal DSA and in 4 cases by clinical observation.

There were performed 71 TR–MRAs and 67 spinal DSAs. In 4 cases with negative TR–MRAs, spinal DSAs were called off. In those cases, the researchers excluded SDAVFs based on medical history, presentation, and clinical course that were unequivocal for lack of spinal vascular malformation.

All imaging studies considered in this systematic review were performed on 1.5-T MR scanners. Table 2 summarizes the other parameters of TR–MRA techniques.

In 42 cases, TR–MRA was true positive, and in 21 cases, it was true negative. We found 7 false-positive cases and 1 false negative (On–line Table 2).

TR–MRA allowed for shunt level identification in 39 cases in total. Of these, the predicted level was correct in 23 cases (59%), to within 1 level in 38 cases (97.4%) and to within 2 levels in 39 cases (100%).

### Meta-analysis results

**Fig. 2:**
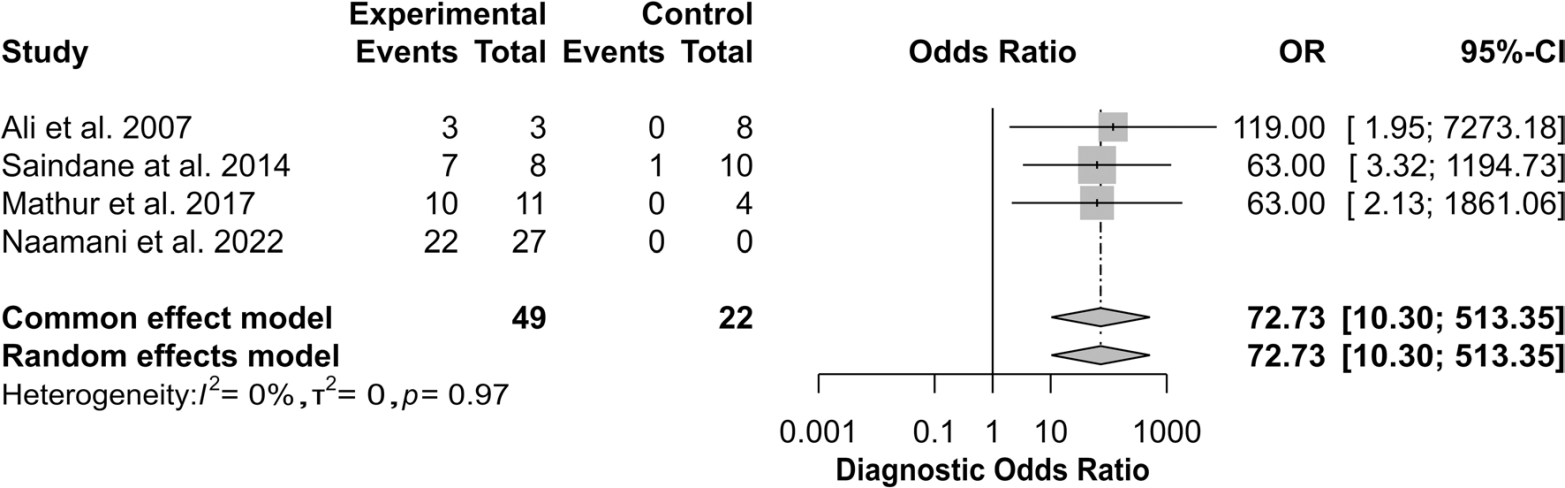
Forest plot of time-resolved MR angiography in spinal dural arteriovenous fistula diagnostic odds ratio. MR, magnetic resonance

**Fig. 3:**
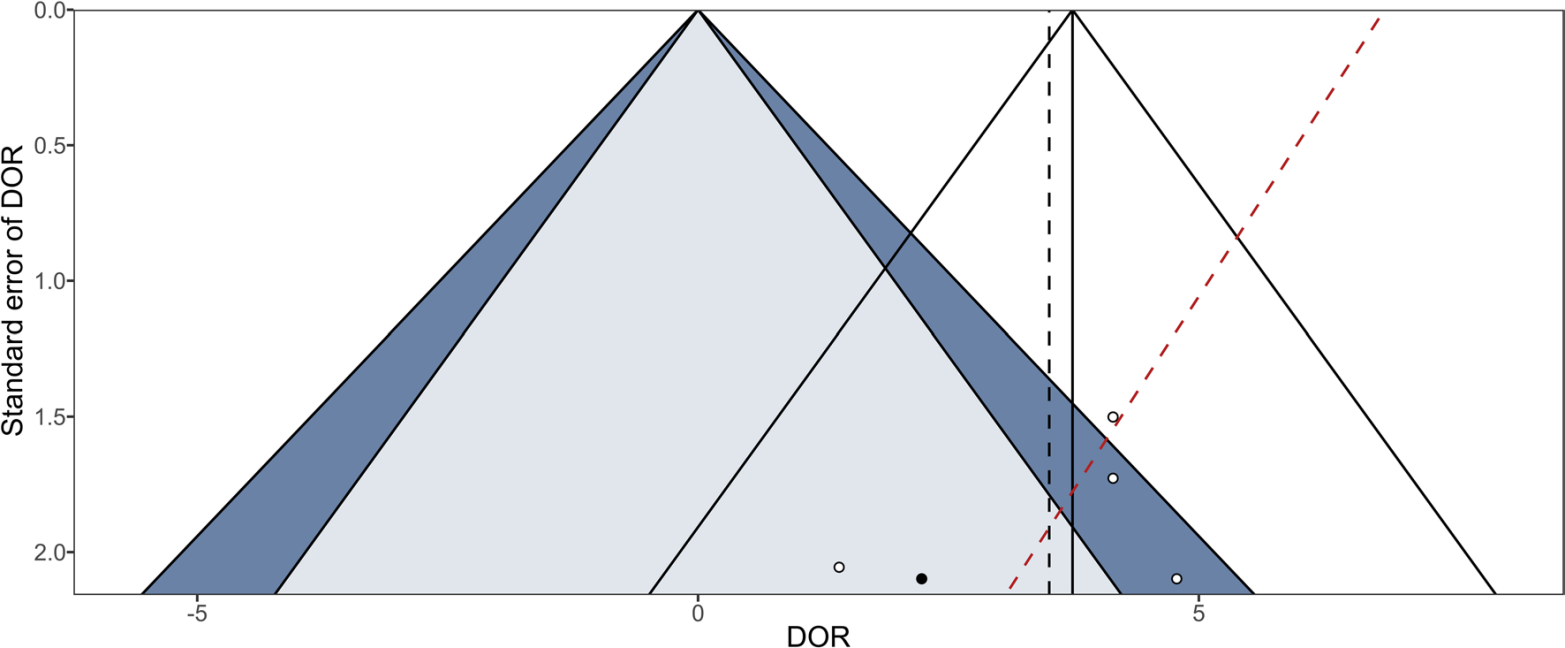
The funnel plot shows publication bias. Standard error of the diagnostic odds ratio (DOR) is plotted against the DOR. DOR is a measure of the effectiveness of a diagnostic test. Markers represent individual studies

**Fig. 4:**
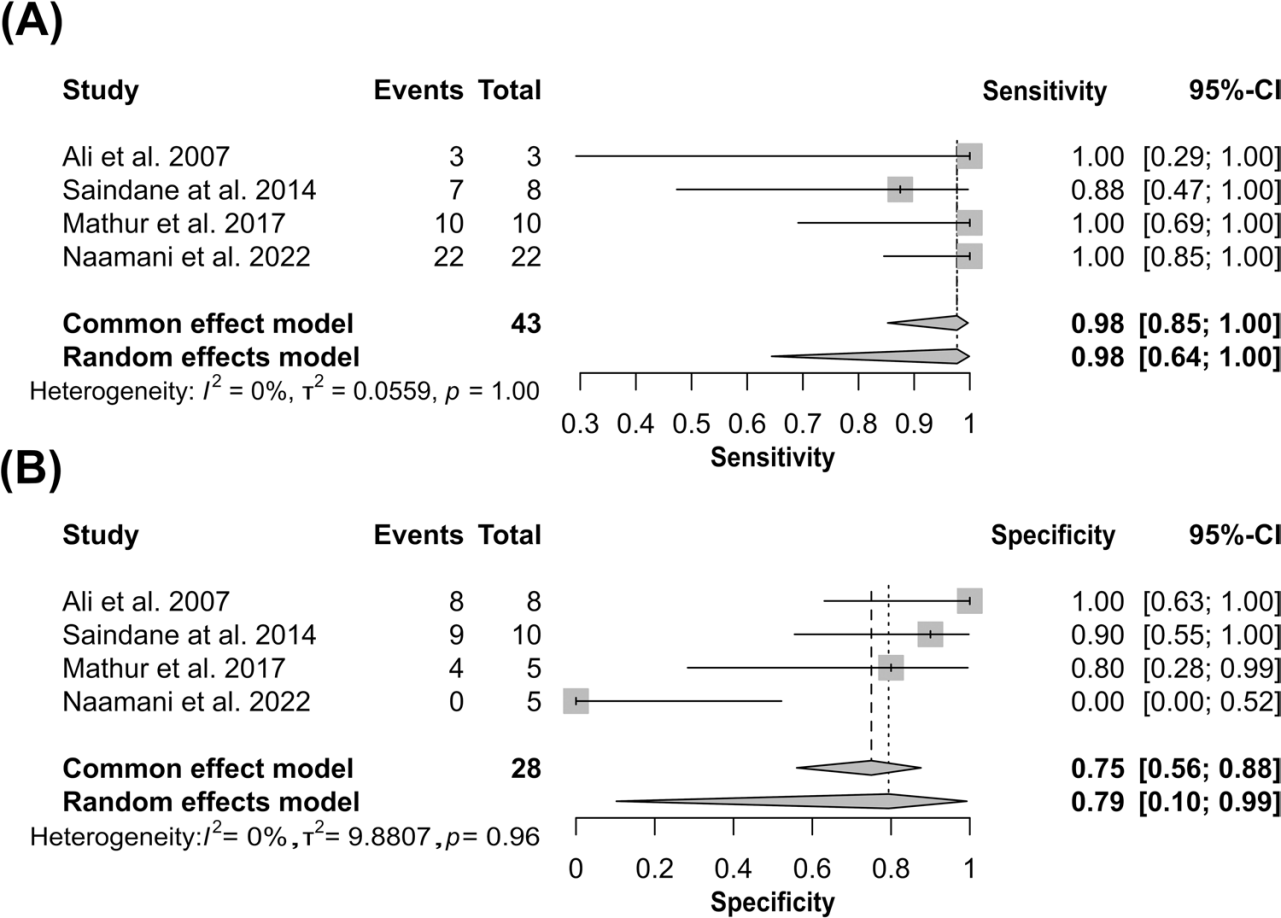
Forest plot of time-resolved MR angiography in spinal dural arteriovenous fistula sensitivity (**A**) and specificity (**B**). MR, magnetic resonance

**Fig. 5:**
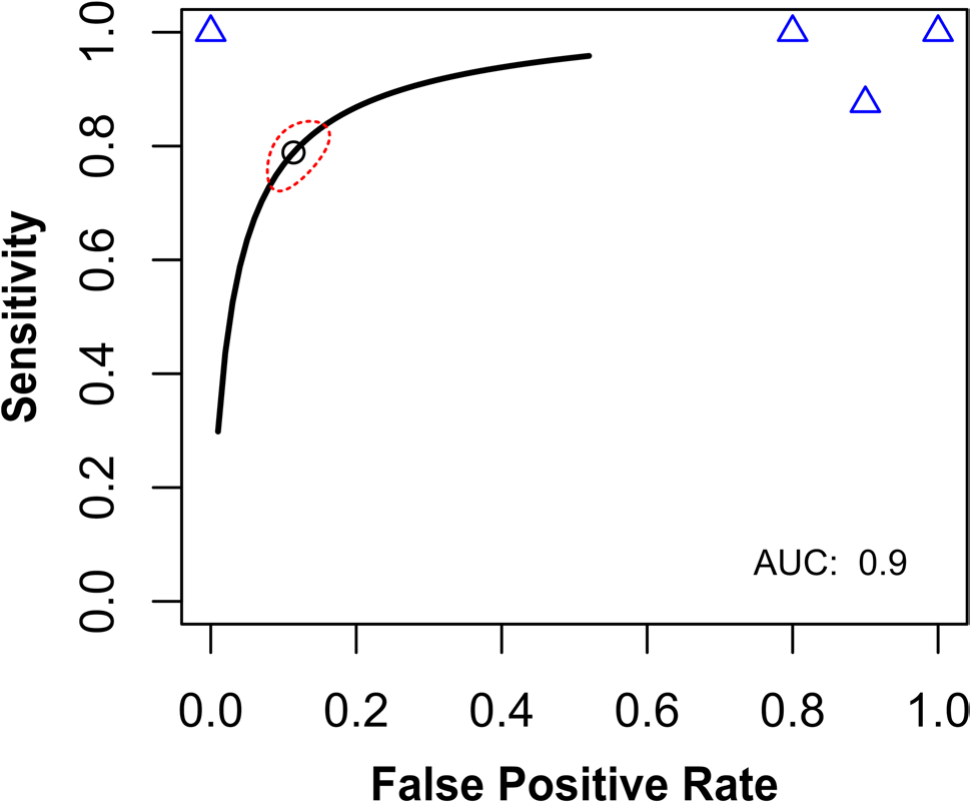
Summary receiver-operating characteristic (SROC) curve showing the relationship between the false-positive rate on the *x*-axis and sensitivity on the *y*-axis. The point estimate of the pair of sensitivity and false-positive rate is shown by a black, open circle. The red, dashed line represents the 95% confidence region. Blue triangles represent input data (results from particular studies)

Four studies reported diagnostic accuracy and provided contingency tables with raw diagnostic accuracy of TR–MRA in SDAVF. The diagnostic odds ratio was 72.73 (95% CI [10.30; 513.35]), *z* = 4.30, *p* value < 0.0001 (Fig. 2). The funnel plot shows publication bias (Fig. 3). The pooled sensitivity was 0.98 (95% CI [0.64; 1.00]) (Fig. 4A), and the pooled specificity was 0.79 (95% CI [0.10; 0.99]) (Fig. 4B). The area under curve (AUC) of the summary receiver-operating characteristic (SROC) curve was 0.9 (Fig. 5).

### Description of included studies

Ali et al. used 1.5-T TR–MRA and spinal DSA to evaluate 11 patients with suspected spinal vascular malformations, 3 of which were SDAVFs [14]. The researchers initially divided patients into high and low suspicion, which was based on presence or absence of early venous shunting in TR–MRA. They inferred level of the shunt through a thorough examination of the TR–MRA. Patients with high suspicion of an arteriovenous fistula underwent spinal DSA, whereas patients with low suspicion either had a spinal DSA or were followed clinically. In cases where TR–MRA was negative, patients were considered true negatives based on clinical outcomes. Subsequent spinal DSAs were planned based on TR–MRA, as described above, with high agreement between the two tests in predicting the location of the shunt. The study reported 100% sensitivity and specificity and no false positives (FPs) or false negatives (FNs).

Saindane et al. evaluated 18 patients using 1.5-T MR, including 8 with SDAVFs [15]. All patients underwent DSA after TR–MRA. Specifically, when TR–MRA was suggestive of a specific arterial feeder, this vessel was catharized first during spinal DSA. When TR–MRA was negative, the spinal DSA was performed in regular fashion. In 4 cases, TR–MRA had limited diagnostic value due to improper scan initiation or patient movements. The sensitivity, specificity, and positive and negative predictive values were 88%, 90%, 88%, and 90%, respectively. TR–MRA matched spinal DSA in 7 cases, with feeding arteries in 6 cases within one vertebral level. In the seventh case, a SDAVF was correctly identified on the TR–MRA, but the feeding artery was not.

Mathur et al. presented 15 patients who had TR–MRA, of which 10 had SDAVF [16]. In all cases, spinal DSA served as a reference test. The researchers first catheterized segmental arteries that were most likely to supply a fistula based on TR–MRA findings. In case the fistula was not identified in such way, the full spinal DSA was performed. The overall sensitivity, specificity, and positive and negative predictive values were 100%, 80%, 91%, and 100%, respectively; all shunt levels were correctly identified to within 1 vertebral level.

Naamani et al. reported 27 patients with SDAVF suspicion, which was subsequently confirmed in 22 cases (20 in spinal DSA and 2 intraoperatively) [17]. In their institution, any patient with an SDAVF suspicion has the spinal DSA following TR–MRA. In 2 cases, the TR–MRA tested positive for SDAVF, contrary to following spinal DSA. Despite that, the vascular malformations were confirmed intraoperatively. Overall sensitivity, specificity, and positive predictive values were 100%, 0%, and 71.4%, while negative predictive value could not be calculated.

Out of the 20 SDAVFs that were positive on both diagnostics tools, 10 (50%) were at the exact level as suggested by the TR–MRA and 10 (50%) were at a different location. With respect to the 10 patients who had discrepancies in their SDAVF localization between the 2 modalities, 9 (90%) patients had a 1-level difference, and 1 (10%) patient had a 2-level difference. Thus, the accuracy of the TR–MRA was 50% in predicting the exact fistula level and 90% accurate in predicting the level correctly to within 1 level.

### Description of excluded studies

Two studies by Kannath et al. and by Amarouche et al. initially had seemed to have met the inclusion criteria but were later excluded.

In a study by Kannath et al. (according to the on-line supplemental diagram), 3 patients from the group with suspected spinal vascular malformation were excluded, because their subsequent spinal DSAs were negative [13]. The authors did not enclose TR–MRA results in these cases, which precludes estimating specificity.

A study by Amarouche et al. lacked the information about false-positive TR–MRAs (what kind of SAVM was suspected) and the results of spinal DSA in cases with false-negative TR–MRAs [12]. Thus, estimating sensitivity and specificity is impossible. The authors tried contacting the researchers but with no success.

### An illustrative case

**Fig. 6:**
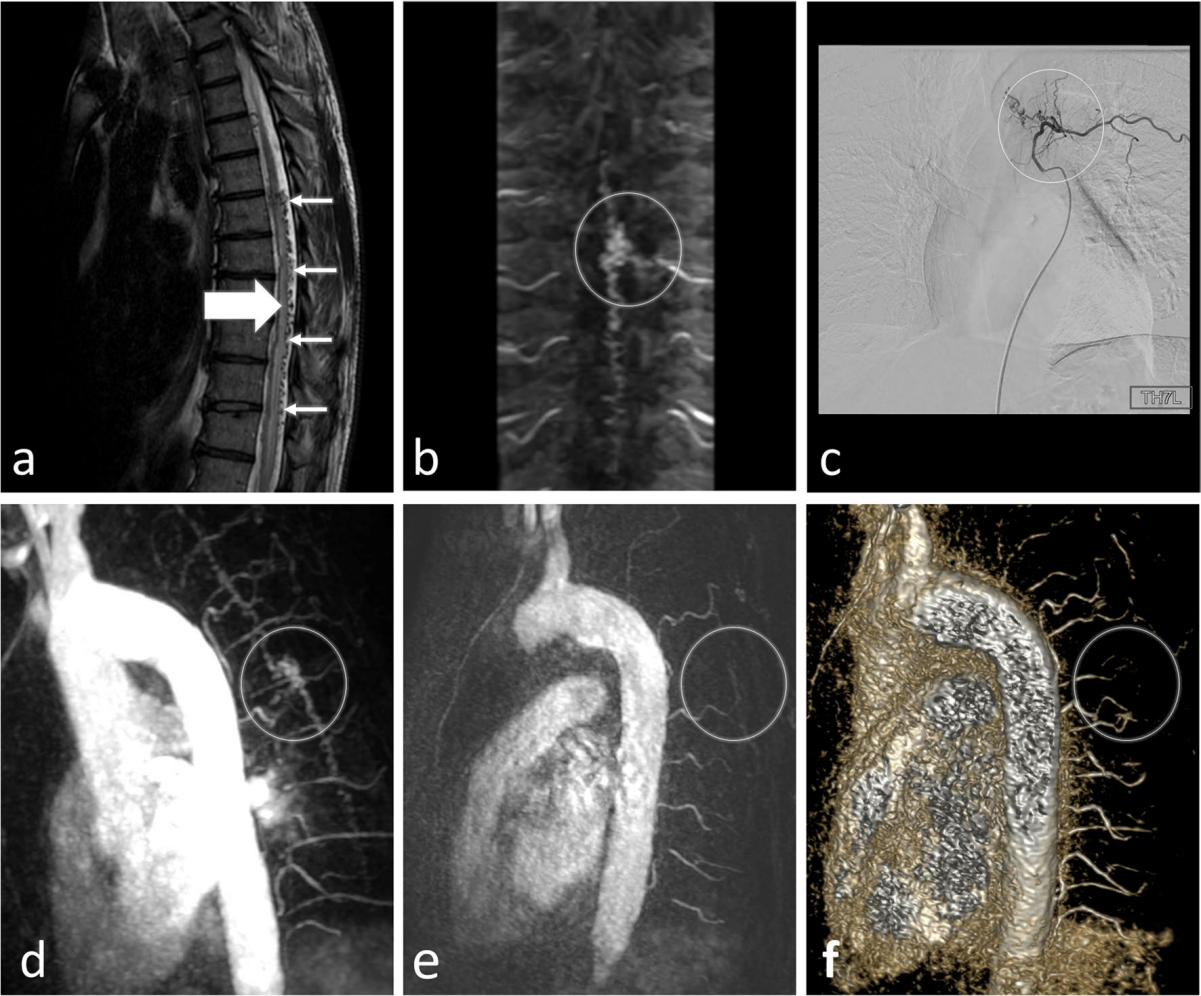
Images of the described illustrative case before (**a**–**d**) and after (**e**, **f**) surgical treatment. An initial sagittal T2-weighted MR image (**a**) displays thickened torturous vessels on the posterior surface of the thoracic spinal cord (**a**, thin arrows) and hyperintensive spinal cord (**a**, big arrow), indicative of myelopathy: these findings are suggestive of spinal dural arterio-venous fistula (SDAVF). A multiplanar reconstruction (MPR) in the coronal plane of time-resolved MR angiography (TR–MRA, **b**) reveals a SDAVF, which is consistent with spinal digital subtraction angiography (DSA, **c**). A maximum intensity projection (MIP) reconstruction in the oblique plane (**d**) before and a subtracted source image (**e**) after the surgery demonstrate the resolution of a SDAVF, further highlighted on the volume rendering (VR) reconstruction (**f**). A circle marks a SDAVF on **b**–**d** and its reduction on **e**, **f**. MR, magnetic resonance

A 61-year-old man presented with a 5-year history of progressive paraparesis of the lower limbs and concomitant urinary disturbance. Upon admission, his neurological examination revealed impaired sensation, spastic lower limb paresis, hyperactive tendon reflexes, and a Babinski sign. Conventional spinal MRI exhibited hyperintensity of the spinal cord in T2-weighted sequences and perimedullary flow voids (Fig. 6).

TR–MRA revealed an early draining and prominent intradural radicular vein, demonstrating a transition from the feeding artery to the arterialized vein at the Th7 level on the left.

This observation was confirmed with spinal DSA. Following the diagnosis of SDAVF, a unilateral hemilaminectomy was performed to surgically disconnect the fistula. Identification and closure of the arterialized vein were achieved, with confirmation provided by intraoperative indocyanine green videoangiography. The patient exhibited favorable tolerance to the procedure. Postoperative TR–MRA did not manifest any residual features indicative of SDAVF. Upon 1-year follow-up, the patient demonstrated significant neurological improvement, achieving unassisted ambulation, albeit with residual minor urinary dysfunction.

## Discussion

Accurately identifying and precisely locating SDAVF are prerequisite for swift and successful surgery. Development of a noninvasive diagnostic tool is a promising idea but requires objective evaluation, which was the reason for this study. In our meta-analysis, we demonstrated that TR–MRA might have sensitivity and specificity in SDAVF detection as high as 98% and 79%, respectively. Based on our results, we believe that TR–MRA has earned the status of and could serve as noninvasive screening study for patients with suspected SDAVFs.

### Spinal DSA limitations

It is worth emphasizing that even though spinal DSA is—and probably will remain—a gold standard, it may mislead in certain cases. In studies by Naamani et al., Przepiorka et al., and Oldfield et al., spinal DSA failed to present SDAVFs that were confirmed intraoperatively [6, 17, 18].

### Advantages and use of TR–MRA

However, there is not enough evidence (nor is the MRI technology advanced enough) to support the notion that TR–MRA should replace spinal DSA. Specifically, it is important to highlight that a negative TR–MRA is not always sufficient to exclude a fistula. Yet, when cautiously interpreted, it may serve as a scout study to facilitate subsequent spinal DSA. As an auxiliary tool, TR–MRA may transform spinal DSA from a long and repetitive diagnostic procedure into a quick confirmatory study with minimal vessels catheterized and reduced iodine contrast and electromagnetic radiation doses.

### TR–MRA findings suggestive for SDAVF

The optimal utilization of the TR MRA technique hinges on the execution of a technically precise examination, facilitating the acquisition of high-quality diagnostic images and their accurate interpretation. Addressing the initial concern, the reduction of the acquisition time for each phase to only 1–2 s enhances temporal resolution, allowing for the imaging of arterial inflow without overlapping veins. Additionally, employing the subtraction technique eliminates static background interference, akin to the principles of DSA, thereby enhancing the visualization of contrasted vessels. Secondly, a meticulous phase-by-phase assessment of both source and reconstructed images assumes paramount importance in the overall evaluation of the study.

After reviewing several research papers, particularly the studies conducted by Mathur et al. and Ali et al., the distinct TR–MRA characteristics and techniques for localizing SDAVFs are adopting their descriptions: (1) a smudge of enhancement in the area of the nerve root dural sleeve that is connected to a branch of the segmental artery; (2) a significant, early draining intradural radicular vein; (3) a morphological transition from feeding arteries to arterialized veins; (4) a cine review of anterograde flow features; and (5) thorough analysis and correlation with unsubtracted source images (as elucidated in the preceding exposition) [14, 16].

The phenomenon known as the “missing-piece sign,” initially elucidated by Zalewski et al. in 2018, visible in most cases in the late phase of the examination, offers valuable assistance in pinpointing the fistula site [19]. This sign manifests as a discrete region of nonenhancement within an extended segment characterized by intense gadolinium enhancement of the spinal cord. It is likely attributed to the variability within the intrinsic venous system of the spinal cord. The segments devoid of enhancement may suggest more effective venous outflow routes compared to the neighboring cord [20].

### Novel multidisciplinary SDAVF diagnosis and treatment algorithm

Based on the results presented herein, we propose the following SDAVF management algorithm that requires effective cooperation between neurosurgeons, MRI radiologists, and interventional radiologists. Firstly, a neurosurgeon should infer the spinal region (as broad as the cervical, thoracic, or lumbosacral region) suspected of SDAVF based on standard spinal MRI and the patient’s clinical presentation. Secondly, MRI radiologists would plan and execute the TR–MRA to locate (or at least approximate) the fistula location. Based on that, an interventional radiologist would precisely diagnose SDAVF with a limited spinal DSA. Finally, a neurosurgeon would be able to disconnect the fistula (or a neurointerventionalist would embolize it) leading to the patient being cured.

### Future directions

Future studies should focus on establishing optimal MRI parameters (such as dose and type of contrast, number of postcontrast phases, temporal update, slice thickness, and features of false-positive results). Additionally, TR–MRA application in other types of spinal vascular malformations, namely, cranial dural arteriovenous fistulas, is yet to be explored.

### Limitations

Studies considered in this systematic review are limited by the small number of patients included. What is more, most of the studies used different MRI protocols. Finally, we could not include one of the biggest series published to date as described in detail above.

Similarly, conclusions from our meta-analysis are limited by a low number of studies included (less than five) and sample sizes (less than 30 patients per group) [21].

## Conclusions

This systematic review and meta-analysis show that TR–MRA may serve as a preliminary study to detect SDAVFs and localize the shunt level with sensitivity and specificity as high as 98% and 79%, respectively. Unless TR–MRA results are unequivocal, it should be followed by limited spinal DSA to confirm SDAVF diagnosis and precisely locate the shunt level. Future studies are needed to further evaluate TR–MRA and advance its development.

## Supplementary Information

Below is the link to the electronic supplementary material.

Supplementary file1 (DOCX 20 KB)

## Data Availability

Data used in this study will be available upon reasonable request should all authors agree.
