# Supplementary material for: Usefulness of time-resolved MR angiography in spinal dural arteriovenous fistula (SDAVF)—a systematic review and meta-analysis
Source: Neurosurg Rev. 2023 Dec 11;47(1):9. doi: 10.1007/s10143-023-02242-7 (PMC10710966; doi:10.1007/s10143-023-02242-7)
Supplement: Supplementary file 1 — Supplementary file1 (DOCX 20 KB) [file 10143_2023_2242_MOESM1_ESM.docx]

|  | **Risk of Bias** | | | | **Applicability Concerns** | | |
| --- | --- | --- | --- | --- | --- | --- | --- |
|  |  |  |  |  |  |  |  |
|  | **Patient selection** | **Index test** | **Reference standard** | **Flow and timing** | **Patient selection** | **Index test** | **Reference standard** |
|  |  |  |  |  |  |  |  |
| Ali et al., 2007^14^ | low | low | high | high | low | low | low |
| Saindane et al., 2014^15^ | high | low | high | low | low | low | low |
| Mathur at al., 2017^16^ | low | low | high | low | low | low | low |
| Naamani at al., 2022^17^ | low | low | unclear | low | low | low | low |

On–line Table 1. Summary of the authors’ judgements about each domain's risk of bias and applicability concerns in the 4 included studies

|  | **TR–MRA** | **DSA** | **TP** | **TN** | **FP** | **FN** |
| --- | --- | --- | --- | --- | --- | --- |
| Ali et al., 2007^14^ | 11 | 7 | 3 | 8 | 0 | 0 |
| Saindane et al., 2014^15^ | 18 | 18 | 7 | 9 | 1 | 1 |
| Mathur et al., 2017^16^ | 15 | 15 | 10 | 4 | 1 | 0 |
| Naamani et al., 2022^17^ | 27 | 27 | 22 | 0 | 5 | 0 |

On–line Table 2. Description of diagnostic tests included in this study. DSA – digital subtraction angiography, FN – false negative, FP – false positive, TN – true negative, TP – true positive, TR–MRA – time resolved magnetic resonance angiography.
